# Characterization and Biomechanical Study of a Novel Magnesium Potassium Phosphate Cement

**DOI:** 10.3390/life12070997

**Published:** 2022-07-05

**Authors:** Zhenchuan Han, Bo Wang, Bowen Ren, Yihao Liu, Nan Zhang, Zheng Wang, Jianheng Liu, Keya Mao

**Affiliations:** 1 Chinese PLA Medical School, Beijing 100853, China; hanzhenchuan301@163.com (Z.H.); docrenbowen@163.com (B.R.); 2 Senior Department of Orthopedics, The Fourth Medical Centre of Chinese PLA General Hospital, Beijing 100089, China; jsdxliuyihao@126.com (Y.L.); wangzheng301@163.com (Z.W.); 3 Department of Orthopedics, Chinese PLA Rocket Force Characteristic Medical Center, Beijing 100088, China; nzhang001@126.com; 4 Department of Orthopedics, Beijing Jishuitan Hospital, Beijing 100035, China; drwangbo@pku.edu.cn

**Keywords:** characterization, cytotoxicity, biomechanical test, magnesium potassium phosphate cement, pedicle screws

## Abstract

Magnesium potassium phosphate cement (MKPC) has attracted considerable attention as a bone regeneration material. However, there are only a few reports on its biomechanical properties. To evaluate the biomechanical properties of MKPC, we compared the mechanical parameters of pedicle screws enhanced with either MKPC or polymethyl methacrylate (PMMA) bone cement. The results show that the maximum pull-out force of the pedicle screws was 417.86 ± 55.57 and 444.43 ± 19.89 N after MKPC cement setting for 30 min and 12 h, respectively, which was better than that of the PMMA cement. In fatigue tests, the maximum pull-out force of the MKPC cement group was 435.20 ± 7.96 N, whereas that of the PMMA cement in the control group was 346.80 ± 7.66 N. Furthermore, the structural characterization analysis of the MKPC cement revealed that its microstructure after solidification was an irregular tightly packed crystal, which improved the mechanical strength of the cement. The maximum exothermic temperature of the MKPC reaction was 45.55 ± 1.35 °C, the coagulation time was 7.89 ± 0.37 min, and the compressive strength was 48.29 ± 4.76 MPa, all of which meet the requirements of clinical application. In addition, the MKPC cement did not significantly inhibit cell proliferation or increase apoptosis, thus indicating good biocompatibility. In summary, MKPC exhibited good biomechanical properties, high initial strength, good biocompatibility, and low exothermic reaction temperature, demonstrating an excellent application potential in the field of orthopedics.

## 1. Introduction

In clinical practice, revision surgeries usually require many autologous bone grafts to fill bone defects. However, this requires destructing normal bone structures elsewhere, which increases the risks of pain and infection [1]. The use of synthetic bone materials eliminates the need to resect bone tissue from a secondary location in the body. Additionally, it does not carry the same risk of disease transmission as allogeneic graft materials [2]. In addition, artificial synthetic bone materials do not suffer from the risks of low supply or high cost associated with autologous bone and allografts. There are also no concerns regarding inconsistencies that can occur because of variability in donor bone quality [3]. Therefore, artificial synthetic bone materials are considered excellent alternatives to autologous and allogeneic bone grafts [4]. Polymethyl methacrylate (PMMA) and calcium phosphate cement (CPC) are widely used clinical bone repair materials [5]. PMMA is one of the most prevalent organic bone cements owing to its excellent mechanical properties and rapid hardening speed. However, it has the inherent drawbacks of toxic organic monomers, high condensation temperature, lack of biocompatibility and degradability, and a strong stress shielding effect [6,7,8]. CPC is the most studied inorganic bone cement, as it has a mineral composition similar to that of native bones, which generates superior biocompatibility and osteoconductivity [9]. However, the drawbacks of a long setting time, poor initial mechanical properties, relatively low degradation rate, and brittleness directly hinder the wide application of CPC [10,11].

Magnesium phosphate cement (MPC) is an inorganic cement developed recently and recognized as a promising candidate for bone cement [12,13,14]. This is due to the outstanding material characteristics of MPC. It exhibits good biocompatibility and degradability, and is absorbed within months in vivo, as demonstrated in animal studies [14,15]. MPC releases magnesium (Mg) ions during its degradation [16]. Magnesium ions play a vital role in mineral metabolism (e.g., calcification, bone density, and hydroxyapatite (HA) crystal formation) and stimulate osteoblast adhesion, proliferation, and stability [17,18]. Moreover, MPC is intrinsically antimicrobial [19]. Although these results provide strong evidence supporting MPC as a promising bone cement, research on MPC remains scarce. In previous studies, MPC was synthesized using magnesium oxide (MgO) as the starting precursor reacting with ammonium phosphate salts [1]. Unfortunately, this produces a potential risk of harmful ammonia generation during cement degradation [16]. To eliminate this risk, magnesium potassium phosphate cement (MKPC) fabricated from MgO and potassium phosphate salts was developed. Fan et al. reported that MKPC and ammonium MPC had similar solidification strengths, and no harmful gases were emitted from MKPC [20]. Moreover, MKPC has a low exothermic reaction temperature. Both improved features showed that MKPC is more suitable for bone-filling materials.

This study aims to evaluate the structural characteristics, biocompatibility, and biomechanical properties of this novel MKPC. In addition to the basic biomechanical study, MKPC cements were tested for their ability to increase the screw stability under osteoporotic conditions, whose objective is to explore the potential of MKPC cements to enhance the stability of orthopedic implants. It was hypothesized that MKPC would provide biomechanical stability equivalent to that of the commercial PMMA cement.

## 2. Materials and Methods

### 2.1. Cement Preparation

**Figure 1:**
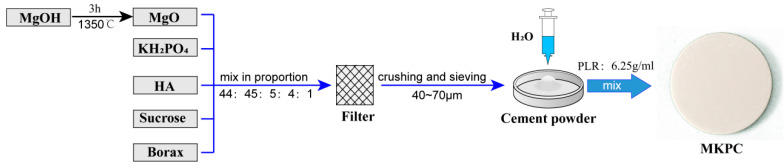
Schematic diagrams of the manufacturing process for magnesium potassium phosphate cement.

Currently, MKPC is not commercially available and was prepared in this work, while a commercially available PMMA cement (Heraeus Corp., Hanau, Germany) was used as the control. MKPC was produced by a mixed reaction of acidic components, alkaline components, curing solutions, and retarders (Figure 1). The alkaline component was magnesium oxide (MgO), which was obtained by calcining magnesium hydroxide (Mg(OH)_2_) at 1350 °C for 3 h. The acidic component was potassium dihydrogen phosphate (KH_2_PO_4_), the curing solution was distilled water, and the retarders were sucrose (C_12_H_22_O_11_) and borax (Na_2_B_4_O_7_·10H_2_O). Additionally, a small amount of hydroxyapatite (HA) was added to the cement. HA was added because studies have shown that mixing HA with other bone materials can effectively improve its mechanical properties and reduce the degradation rate of the material [21]. Subsequently, MgO, KH_2_PO_4_, HA, sucrose, and borax were ground and sifted, and a 40–70 μm powder was selected for the preparation of the cement. The cement powder was obtained by mixing MgO:KH_2_PO_4_:HA:sucrose:borax at a ratio of 44:45:5:4:1, which was blended with distilled water in a powder-to-liquid ratio of 6.25 g/mL to obtain MKPC. The detailed composition and method of preparing MKPC were based on previous research [1,5,13,22]. Lastly, this study analyzed a possible clinical application prospect of MKPC, which was facilitated by Ningbo Hairen Biotechnology Co., Ltd. (Ningbo, China).

### 2.2. Compressive Strength, Exothermic Temperature, and Setting Time

The cement pastes were transferred into silicone rubber molds (6 × 6 × 12 mm^3^) (Beijing Institute of Medical Device Testing, Beijing, China), stored for 1 h at 37 °C and >90% humidity, demolded, covered with phosphate-buffered saline (PBS pH = 7.4), and stored at 37 °C. The wet compressive strength of the cuboids (*n* = 6) was measured 24 h after fabrication. A universal testing machine, Instron3365 (Instron Corp., Norwood, MA, USA), was used at a crosshead speed of 1 mm/min. The temperature change during the setting of the cement was measured every 15 s by a laser temp-gun thermometer (OS52, OMEGA, Hartford, CT, USA). The measurement was stopped when the setting cement returned to room temperature (23 ± 1 °C). The final setting times of the cement samples were determined using a Gillmore needle (Humboldt Mfg. Co., Frankfurt, Germany) at room temperature, following ISO 9917. The final setting time was recorded in minutes when the heavy needle failed to leave an indentation deeper than 1 mm on the cement surface. For statistical purposes, six specimens were tested for each type of sample.

### 2.3. XRD and SEM

The hardened bone cement was ground into powder, and the chemical composition was characterized using X-ray diffraction (XRD; Siemens D5005, Berlin, Germany). XRD was performed using Cu-Kα radiation, 40 kV voltage, 40 mA current, 2 theta range from 10° to 70°, step size of 0.02°, and a scan rate of 1.5 s/step. For the qualitative analysis, the measurement curves were compared with Joint Committee on Powder Diffraction Standards (JCPDS) reference curves. The bone cement was dried, the surface of the sample was sprayed with gold, and the surface morphology of the sample was observed using scanning electron microscopy (SEM, FEI, Quanta250, Hillsboro, OR, USA) with an operating voltage of 5 or 12.5 kV. To analyze the interdigitation of bone cement into the adjacent spongiosa in the screw channel, a cross-sectional cut screw channel was prepared. Subsequently, images of the exposed surfaces were obtained using SEM.

### 2.4. In Vitro Cytotoxicity Test

Human osteosarcoma cells (MG-63) were cultured in a humidified incubator. The cells were harvested at the confluence with 0.25% trypsin and seeded onto the disks with an initial density of 2000 cells per well in a 96-well plate and incubated at 37 °C/5% CO_2_. MKPC extracts were prepared according to procedures reported in the literature [23]. First, solutions were obtained by adding sterilized cement to serum-free DMEM at a solid/liquid ratio of 0.2 g/mL. After incubation at 37 °C for 24 h, the mixture was centrifuged, and the supernatant was collected and then stored at 4 °C until further use. The cytotoxicity of MPC was evaluated using a Cell Counting Kit-8 (CCK-8, Dojindo, Kumamoto-ken, Japan). The optical density (OD) of the solution was measured at 450 nm using a microplate reader at 24, 48, and 72 h. MG-63 cells were co-cultured with the cement extract solution for 24 h. The cells were trypsinized and centrifuged at 1000 rpm for 5 min. Subsequently, they were incubated with cell surface marker-specific antibodies (Annexin V-FITC, C1063, Beyotime, Shanghai, China) for 15 min, rinsed with PBS and centrifuged at 1000 rpm for 5 min to remove the supernatant. Apoptosis and cell death were analyzed using flow cytometry.

### 2.5. Biomechanical Tests

In this study, standardized Sawbone modules (Pacific Research Laboratories Inc., Vashon Island, WA, USA) were used for biomechanical testing, and cadaver vertebrae were used only for verification to ensure the reliability of the experimental data. This method reduced the dependence on human cadaver vertebrae and the experimental error caused by differences in individual vertebrae. The lowest-density Sawbones modules (Sawbones 5^#^ = 0.07~0.08 g/cm^3^, compressive strength = 0.45~0.78 MPa) from ASTM F1839-08 were used to simulate severe osteoporotic conditions. The minimum density of Sawbone modules simulates the condition of human spinal bone with severe osteoporosis, owing to its extremely porous and permeable characteristics [24]. At the same time, owing to the spongy structure of the Sawbones, less pressure was required to inject cement without special equipment, thereby reducing the cost of the experiment. Five lumbar spine specimens were used for biomechanical testing. Radiographic screening was performed for all lumbar vertebrae to exclude fractures, tumors, or other conditions that could affect biomechanics. Sawbone modules and lumbar specimens were carefully drilled to prepare the screw channel (Φ 5.0 × 40 mm). The Sawbone modules were divided into two groups: the test group was injected with 1 mL MPC in the screw channel, and the control group was injected with 1 mL PMMA in the screw channel. Similarly, one side of the lumbar vertebrae was randomly selected for injection with 1 mL MPC in the screw channel, and the contralateral lumbar vertebrae were injected with 1 mL PMMA in the screw channel. Then, pedicle screws (Φ 6.0 × 40 mm, IRENE Corp., Tianjin, China) were appropriately tightened by a single investigator to reduce variations in insertion torque. The reinforced pedicle screws were subjected to pull-out, torsion, and fatigue tests using a material-testing machine. The grouping and mechanical testing of the samples tested in this study are presented in Table 1.

**Figure 2:**
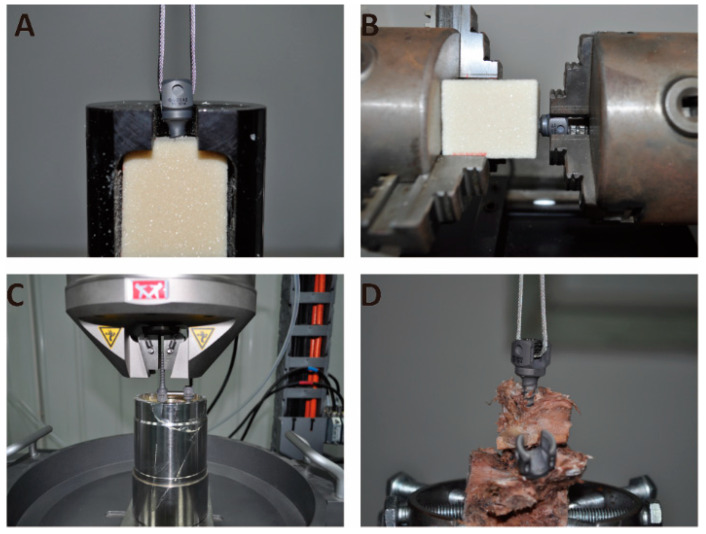
(**A**) Screw pull-out test setup. (**B**) Screw torsion test setup. (**C**) Screw fatigue test setup. (**D**) Screw pull-out test device in vertebral body.

Pull-out test (Figure 2A,D): A pre-load of 50 N was applied to prevent a sudden impact that may occur at the initiation of the pull-out test. A continuous and progressive load was applied at a speed of 5 mm/min to pull out the pedicle screw and record the peak pull-out force (PPF). Torsion testing (Figure 2B): The orientation of pedicle screws was adequately adjusted. The pedicle screws were rotated anticlockwise at 5°/min. The peak torque (N/mm) was calculated, indicating the holding power of the screw–bone–cement interface. Fatigue testing (Figure 2C): To evaluate the effect of cement on the stability of pedicle screws under cyclic physiological loading, a non-destructive cyclic load (1 Hz, 75 N, 20,000 cycles) was applied to the titanium rod fixed to the screw [25]. The PPF of the pedicle screws was then tested.

### 2.6. Statistical Analysis

All results are presented as the mean ± standard deviation. A normal distribution was confirmed, and significant differences were calculated using a one-way ANOVA. Non-normally distributed data were analyzed using the Kruskal–Wallis test, followed by the Mann–Whitney U-test to find significant differences between groups. Statistical analyses were performed using GraphPad Prism^®^ (7.04, GraphPad Software Inc., La Jolla, CA, USA).

## 3. Results

### 3.1. Material Characterization

The compressive strength, exothermic temperature, and setting time of MKPC are summarized in Table 2. In this study, the compressive strength of MKPC was 48.29 ± 4.76 MPa, which was significantly higher than the 35 MPa of CPC reported in the literature [26] and lower than 70 MPa of PMMA cement (ASTM F451-08). The maximum temperature of MKPC was 47.50 °C, which was also significantly lower than the maximum temperature of 90 °C for PMMA cement (ASTM F451-08). The setting time of MKPC was 7.89 ± 0.37 min, which provided a suitable time for clinicians to operate.

**Figure 3:**
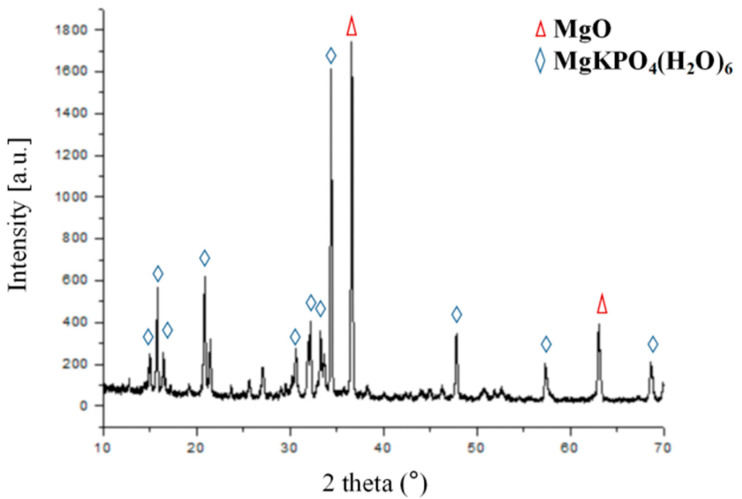
X-ray diffraction patterns of the MKPC samples after setting for 24 h. The phases were identified according to the following PDF numbers: MgKPO_4_(H_2_O)_6_ (JCPDS PDF: 75-1076) and MgO (JCPDS PDF: 45-0946).

**Figure 4:**
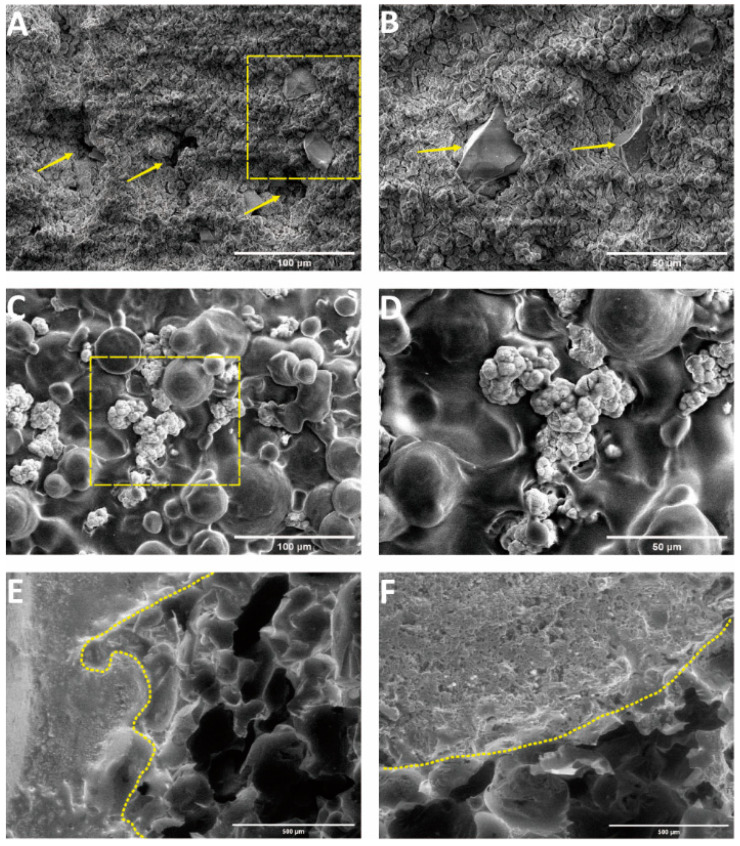
SEM images of hardened cement at different magnifications. (**A**) The MKPC cement exhibited a microstructure with the interlocked flaky crystals and micropores; (**B**) The excess unreacted MgO particles were embedded in the MKPC cement; (**C**,**D**) The PMMA cement was in the form of dense xerogel with dispersed fine particles; (**E**) The MKPC cement was embedded into the Sawbones pores and achieved an anchoring effect; (**F**) The PMMA cement was not well embedded in the Sawbones pores, and the anchoring effect is insufficient.

After 24 h of hardening of MKPC, XRD analysis was carried out using an X-ray diffractometer to determine the solid content (Figure 3). Some of the diffraction peaks in the diffraction pattern of MKPC were consistent with the characteristic diffraction peaks of the PDF standard card (JCPDS PDF:75-1076) of potassium magnesium phosphate (MgKPO_4_·6H_2_O), and some of the diffraction peaks were consistent with the characteristic diffraction peaks of the PDF standard card (JCPDS PDF:45-0946) of MgO. Because of the non-stoichiometric mixing ratios between the cement powder and liquid, the reaction was not quantitative, and the end-product (MgKPO_4_·6H_2_O) coexisted with excess unreacted MgO. The surface micro-morphologies of MKPC, PMMA, and the interface between the cement and sawbones were observed using a scanning electron microscope. After setting, the MKPC cement exhibited a microstructure with interlocked flaky crystals and high porosity (Figure 4A,B), which is a typical MPC crystal structure. The microstructure of MKPC can provide mechanical support and induce the growth of new bone. As shown in Figure 4B, the unreacted MgO particles were embedded in the MKPC cement. The PMMA cement was in the form of a dense xerogel with fine dispersed particles (Figure 4C,D). It was observed that MKPC was well embedded into the Sawbones pores and achieved a good anchoring effect (Figure 4E), but the PMMA cement was not satisfactorily embedded into the Sawbones pores (Figure 4F).

**Figure 5:**
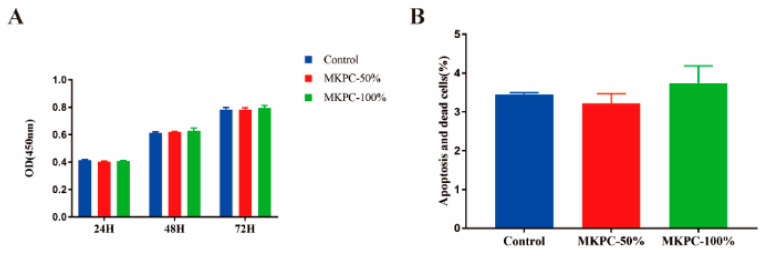
Effects of the MKPC cement on cell proliferation and apoptosis in vitro. (**A**) The results of the CCK-8 experiment provide the cell proliferation in different concentrations of extracts, and the results were not statistically different (*p* > 0.05). (**B**) The cell was co-cultured with cement extract solution for 24 h, with no significant difference in apoptosis and death percentage (*p* > 0.05).

Figure 5A shows the proliferation of MG63 cells cultured in the extract solution of MKPC for 24 h, 48 h, and 72 h. The optical density (OD) values from the CCK-8 assay provided an indicator of cell growth and proliferation at various concentrations of the extract solution (50% and 100%). It can be seen that cell proliferation on various concentrations of the extract solution increased with time, with no significant differences (*p* > 0.05). Moreover, Mg-63 cells co-cultured with the cement extract solution for 24 h had no significant difference in apoptosis and death percentage (Figure 5B: *p* > 0.05). These results indicate that the MKPC cement caused no cytotoxicity.

### 3.2. Biomechanical Tests

**Figure 6:**
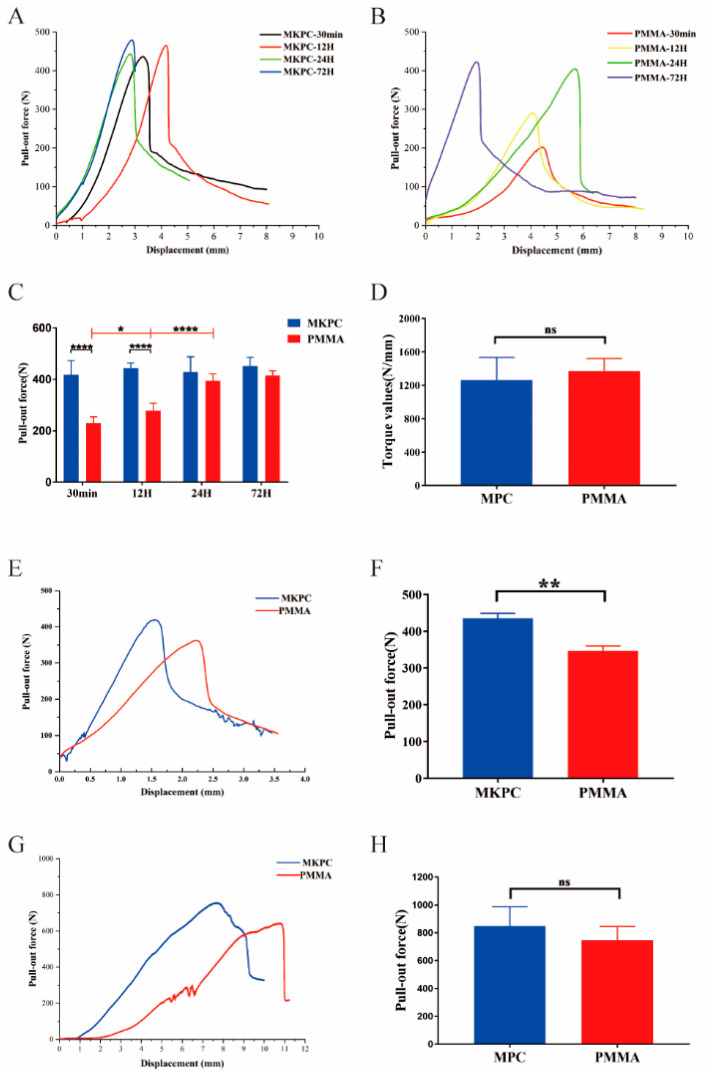
In vitro biomechanical study of screw strengthening effect of MKPC cement. (**A**) The load–displacement curve of the pull-out force of the MKPC cement-reinforced screws was similar at different time points of cement curing. (**B**) The pull-out resistance of the PMMA cement-reinforced screws gradually increased with time, and the mechanical curve tended to be stable after the cement curing for 24 h. (**C**) The pull-out resistance of MKPC cement-reinforced screws was better than that of PMMA cement at 30 min and 12 h, and the two tended to be similar after 24 h. (**D**) There was no significant difference between the two types of bone cement in the torsion resistance of reinforced screws (*p* > 0.05). (**E**) Load–displacement curve after cyclic loading of the screw. (**F**) After cyclic loading of the screws, the pull-out resistance of the screws strengthened with MKPC cement was significantly better than that of the PMMA cement group (*p* < 0.01). (**G**) Load–displacement curves of pull-out resistance of the cement-enhanced pedicle screws in the vertebral body. (**H**) There was no statistical difference between the two kinds of bone cement in the anti-pulling ability of the screw in the vertebral body (*p* > 0.05). (ns: *p* > 0.05; *: *p* < 0.05; **: *p* < 0.01; ****: *p* < 0.0001).

First, the pedicle screw pull-out force test was performed, and the screw pull-out force was measured according to different cement curing times, as shown in Figure 6A–C. The screw can obtain a stable holding force after the MKPC cement solidified for 30 min (Figure 6A). The time for which the cement solidified (30 min to 72 h) did not significantly change the screw pull force curve. As shown in Figure 6C, the maximum pull-out force of pedicle screws was 417.86 ± 55.57, 444.43 ± 19.89, 428.70 ± 59.56, and 451.79 ± 34.40 N after MKPC cement setting for 30 min, 12 h, 24 h, and 72 h, respectively. There was no significant difference in the maximum pull-out force of the MKPC cement at different time points after setting (*p* > 0.05). As shown in Figure 6B, the holding force of the PMMA cement on the pedicle screws gradually increased over time, and the mechanical properties of the cement tended to stabilize after setting for 24 h. Figure 6C shows that the maximum pull-out force of the pedicle screws measured at 30 min, 12 h, 24 h, and 72 h of the PMMA cement setting were 231.19 ± 23.13, 278.05 ± 29.30, 395.16 ± 27.14, and 415.50 ± 18.57 N, respectively. There was a statistically significant difference between the maximum pull-out forces at 30 min, 12 h, and 24 h (*p* < 0.05). The maximum pull-out force of the MKPC cement on pedicle screws was better than that of the PMMA cement at 30 min, 12 h, and the two were more similar after 24 h.

Because the PMMA cement stabilizes after curing for 24 h, the torsion test, fatigue test, and vertebral body test were selected when the cement was cured for 24 h (Figure 6D–H). Figure 6D shows that there was no significant difference in the maximum torsion force (N/mm) between the MKPC cement and PMMA cement in the torsion test (*p* > 0.05). To further evaluate the ability of the cement to enhance the screw pull-out resistance in a dynamic environment, fatigue tests were performed, and the results are shown in Figure 6E,F. The maximum pull-out force of the MKPC cement group was 435.20 ± 7.96 N, whereas that of the PMMA cement in the control group was 346.80 ± 7.66 N. There was a statistically significant difference in the maximum pull-out force between the two groups after dynamic loading (*p* < 0.01). To further evaluate the mechanical properties of bone cement in human bone, the maximum pull-out force of cement-reinforced pedicle screws in the vertebral body was tested (Figure 6G,H). After 24 h of cement setting, the maximum pull-out force of the MKPC cement group was 849.1 ± 61.56 N, whereas that of the PMMA cement in the control group was 745.9 ± 44.57 N. The two groups showed similar maximum pull-out forces with no statistical significance (*p* > 0.05).

## 4. Discussion

Bone cement is used clinically to improve the stability of internal fixation implants [27]; therefore, the mechanical properties play important role in the clinical application of bone cement. MPC bone cement is a mixture of magnesium oxide, phosphate, retarder, and curing liquid [16]. The properties and ratios of these components determine the physicochemical properties of MPC. Ammonium magnesium phosphate-based cement is a widely studied cement formulation [1]. However, the ammonia released by these cements may compromise their biocompatibility [28], which has resulted in the magnesium ammonium phosphate cement becoming less popular. This study demonstrates that a novel potassium salt type magnesium phosphate cement can be generated using low-activity magnesium oxide and potassium dihydrogen phosphate. The use of potassium dihydrogen phosphate to replace the conventional acidic component ammonium dihydrogen phosphate to synthesize the MPC system bone cement eliminates the burden of NH_3_ produced by the MPC system bone cement after curing and potentially increases the biocompatibility of the MPC bone cement. The cement components used in this study were MgO + KH_2_PO_4_ + 5H_2_O → MgKPO_4_·6H_2_O (Figure 1). Borax was used as a retarder to slow the reaction rate and prolong the setting time of cement [29]. The addition of hydroxyapatite makes the microstructure of magnesium phosphate cement more compact, reduces the appearance of gaps, and increases the mechanical strength of the cement [9].

The MKPC reaction proceeded at room temperature (23 ± 1 °C), and the exothermic temperature was only 45.55 ± 1.35 °C for the setting of 10 g cement powder with 1.6 mL deionized water. Bone necrosis occurs at 50 °C and protein denaturation occurs at 56 °C [30], well above this temperature. The polymerization temperature of MKPC is much lower than that of PMMA cement (approximately 51.20 ± 6.20–86.70 ± 10.70 °C) [31,32]. The compression strength of MKPC in this study was in the range of approximately 43.10–56.04 MPa. The compressive strength of MKPC was lower than that of PMMA but better than that of traditional CPC, and can be used to repair some non-load-bearing bones. MPC cement has been studied in various animal models and defects, including cranial defects in rabbits, securing dental implants in dogs, and metacarpal and metatarsals in horses [33,34,35], which have demonstrated improved adhesion and healing in nearly all surgical sites. All strengths were measured after 24 h of setting and incubating the samples in PBS buffer without drying them before testing; thus, the strength values represented the clinically attainable mechanical properties. The MKPC cement provided the clinically acceptable setting times of 7.89 ± 0.37 min, which were in the same range as those obtained for a commercial PMMA cement (Heraeus OSTEOPAL^®^ V, hardening time of 8 min is given by the manufacturer at 23 °C) [36]. The X-ray diffraction analysis of the cement after setting revealed that the main products of MKPC were MgKPO_4_·6H_2_O and MgO. As the cement component is based on the mass ratio instead of the chemical reaction ratio, excessive unreacted MgO remained after the reaction. Observing the microstructure of the MKPC material after curing (Figure 4A,B), it is seen that MKPC is composed of irregular closely bonded crystals, with pores are scattered on the surface. The tightly bonded crystal structure promotes the mechanical strength of MKPC bone cement to a certain extent. There is a pore-like structure between MKPC crystals, which provides sufficient space for the ingrowth of new bone, induces bone ingrowth, increases the contact area between the bone cement material and new bone tissue, and enhances the adhesion between bone cement and bone. The microstructure of the PMMA cement after curing (Figure 4C,D) is a dense xerogel with no pores on the surface, and high mechanical strength due to this dense microstructure. By observing the contact surface of the cement and polyurethane foam (Figure 4E,F), it is found that, compared with the PMMA cement, MKPC can better penetrate the cavity of polyurethane foam and achieve a good anchoring effect. This may be due to the smaller crystal structure of MKPC, which makes it easier to embed it into adjacent structures. In this study, the biosafety of MKPC bone cement was verified using in vitro cell proliferation and apoptosis assays (Figure 5A,B). The results show that the MKPC cement did not significantly inhibit cell proliferation or increase apoptosis, while exhibiting good biocompatibility.

Sawbone modules are often used to simulate cancellous bone in biomechanical research [37]. Compared to cadaveric bones, Sawbones modules have a uniform density and structure, which avoids errors caused by the cadaver test materials. The results of the biomechanical study show that the maximum axial pull-out forces of the screw measured when the cement was cured for 30 min and 12 h after reinforcement with MKPC bone cement were significantly better than that of the PMMA bone cement. It was found that, when MKPC bone cement was cured for 30 min, the strengthening performance of the screws was stable, and the average maximum pull-out force reached 97% of the average maximum pull-out force when the cement was cured for 24 h, which was close to the final strength. This shows that MKPC bone cement solidifies quickly and that good mechanical properties can be obtained at an early stage. However, with an increase in the curing time, the maximum axial pull-out force of the PMMA bone cement gradually increased, and the mechanical properties of the cement tended to be stable when the cement was cured for 24 h at 58% of the maximum pull-out force. These results suggest that MKPC bone cement is superior to the PMMA bone cement in terms of rapid setting and high initial strength, especially within 24 h. This is the first time that this result has been reported. In the torsion tests, the MKPC and PMMA cements exhibited similar resistance to screw rotation. All tested screws showed separation of the cement and screw interface, but no detachment of the cement and Sawbones module interface. This is because the cement penetrates the pores of the Sawbones modules to form several anchoring structures compared to the smooth metal contact surface. This significantly increases the friction between the cement and the adjacent Sawbones interface. To simulate the real force of the screw in the body, the screw was subjected to a complex mechanical environment by applying a cyclic load to the end of the titanium rod. The results show that the maximum pull-out force of the screw reinforced with MKPC bone cement was still significantly greater than that of the PMMA bone cement, indicating that the MKPC bone cement had better resistance to complex mechanical environments. This is because the MKPC bone cement can better form an anchoring structure with the interface of Sawbones modules (Figure 4E). In the cement-reinforced intravertebral screw pull-out test, the MKPC cement and PMMA cement exhibited similar mechanical properties (Figure 6H). The experimental results are in good agreement with the results measured in the Sawbones module, which further verified the mechanical reliability of the MKPC cement.

However, this biomechanical experiment has several limitations. Firstly, applied loading may not exactly represent what occurs in clinical practice. Although pure pull-out is not the failure mode seen in clinical situations, pull-out testing is still considered a good predictor of pedicle screw fixation strength [38]. Secondly, the biomechanical environment in the current study cannot be completely reconstructed as a real physiological situation; however, this experiment provides considerable information for reference.

## 5. Conclusions

In the present research, the structural characteristics, biocompatibility, and biomechanical properties of the novel MKPC bone cement were evaluated. The results show that the MKPC bone cement is superior to the PMMA bone cement in terms of rapid setting and high initial strength, especially in 24 h. In addition, MKPC had better resistance to complex mechanical environments than PMMA. The structural characterization analysis of the MKPC cement showed that the tightly bonded crystal microstructure is responsible for its high mechanical strength. Furthermore, MKPC also exhibited a low reaction exothermic temperature, suitable curing time, and low cytotoxicity. These characteristics of the new MKPC cement make it highly suitable for potential applications in the field of orthopedics.

## Figures and Tables

**Table 1:** Biomechanical test group.

Test Grouping	Cement Setting Time			
	30 min	12 h	24 h	72 h
Pull-out testing (*n* = 6)	M/P	M/P	M/P	M/P
Torsion testing (*n* = 6)			M/P	
Fatigue testing (*n* = 6)			M/P	
Vertebral body testing (*n* = 5)			M/P	

M: pedicle screws with MKPC. P: pedicle screws with PMMA cement.

**Table 2:** The compressive strength, exothermic temperature, and setting time of the MKPC.

Samples (*n* = 6)	1	2	3	4	5	6	Mean ± SD
Compressive strength (MPa)	56.04	43.10	46.06	44.52	50.76	49.23	48.29 ± 4.76
Exothermic temperature (°C)	45.20	43.70	47.50	44.90	46.70	45.30	45.55 ± 1.35
Setting time (min)	7.58	7.83	8.50	8.17	7.58	7.67	7.89 ± 0.37

## Data Availability

The data that support the findings of this study are available from the corresponding author upon reasonable request.
